# Soft TCPTP Agonism—Novel Target to Rescue Airway Epithelial Integrity by Exogenous Spermidine

**DOI:** 10.3389/fphar.2016.00147

**Published:** 2016-06-03

**Authors:** Carlo A. Ghisalberti, Rosa M. Borzì, Silvia Cetrullo, Flavio Flamigni, Gaetano Cairo

**Affiliations:** ^1^Department of Biomedical Sciences for Health, University of MilanMilan, Italy; ^2^TixupharmaMilan, Italy; ^3^Laboratory of Immunorheumatology and Tissue Regeneration, Rizzoli Orthopaedic InstituteBologna, Italy; ^4^Department of Biomedical and Neuromotor Sciences, University of BolognaBologna, Italy

**Keywords:** TCPTP, spermidine, chronic airway disorders, FGF, TGF, stress growth factors, epithelial to mesenchymal transition, autophagy

## Abstract

A reparative approach of disrupted epithelium in obstructive airway diseases, namely asthma and chronic obstructive pulmonary disease (COPD), may afford protection and long-lasting results compared to conventional therapies, e.g., corticosteroids or immunosuppressant drugs. Here, we propose the polyamine spermidine as a novel therapeutic agent in airways diseases, based on a recently identified mode of action: T-cell protein tyrosine phosphatase (TCPTP) agonism. It may include and surpass single-inhibitors of stress and secondary growth factor pathway signaling, i.e., the new medicinal chemistry in lung diseases. Enhanced polyamine biosynthesis has been charged with aggravating prognosis by competing for L-arginine at detriment of nitric oxide (NO) synthesis with bronchoconstrictive effects. Although excess spermine, a higher polyamine, is harmful to airways physiology, spermidine can pivot the cell homeostasis during stress conditions by the activation of TCPTP. In fact, the dephosphorylating activity of TCPTP inhibits the signaling cascade that leads to the expression of genes involved in detachment and epithelial-to-mesenchymal transition (EMT), and increases the expression of adhesion and tight junction proteins, thereby enhancing the barrier functionality in inflammation-prone tissues. Moreover, a further beneficial effect of spermidine may derive from its ability to promote autophagy, possibly in a TCPTP-dependent way. Since doses of spermidine in the micromolar range are sufficient to activate TCPTP, low amounts of spermidine administered in sustained release modality may provide an optimal pharmacologic profile for the treatment of obstructive airway diseases.

## Introduction

Asthma and chronic obstructive pulmonary disease (COPD) are widespread respiratory disorders, both responding to bronchodilator drugs and to a reduction of toxic insults, e.g., smoking cessation. However, lung function is fully reversible only in subjects with asthma, whilst COPD shows a steady progress pattern. Both asthma and COPD are inflammatory disorders, but their inflammatory patterns differ: the mast-cell/CD4+ T cells/eosinophils axis prevails in asthma, whereas neutrophils/CD8+ T cells (Barnes, 2008) and premature lung senescence (Chilosi et al., 2013) associated to the activation of the receptors of advanced glycation end-products (RAGE; Robinson et al., 2012) fuel COPD. Indeed, most treatments for COPD and asthma are presently focused on suppressing the inflammatory cascades. However, the two conditions share other similarities, such as excessive mucus secretion, impaired epithelial barrier, and obstructive condition. Although the integrity of impaired airways is deemed to be a key player in initiating and determining the clinical outcomes in respiratory diseases (Tam et al., 2011), little attention is paid to its control.

The expression of genes that encode junctional proteins, such as claudin-1 and zona occludens (ZO)-1, and adherent junctional E-cadherin is repressed in “leaky” airways (Balda and Matter, 2009; Pohl et al., 2009; Shaykhiev et al., 2011). In turn, defective tight junctions allow the penetration of further toxic insults. The susceptibility to environmental injury leads to the persistent T_*H*_2-activation of lymphoid cells, whose interplay with myeloid cells results in the increase of circulating and intra-cellular levels of transforming growth factor (TGF)-β.

Stressor autocrine pathways induced by TGF-β upregulation lead to epithelial-to-mesenchymal transition (EMT; Grainge and Davies, 2013), i.e., the loss of epithelial phenotype and the acquisition of mesenchymal phenotype, which plays an important role in the airway remodeling that occurs in chronic bronchial diseases (Pain et al., 2014). Aberrant epithelium repair prompts the accumulation of fibroblasts which initiate the deposition of extracellular matrix (ECM), thus sustaining persistent bronchial obstruction.

Other cytokines, such as tumor necrosis factor (TNF)-α, via Src kinases activation, concur to determining the barrier dysfunction observed in chronic airways diseases (Hardyman et al., 2013). The consequence is a chronic, self-sustained wound scenario dominated by the secretion of secondary stress growth factors (Heijink et al., 2012; Gan et al., 2013; Hardyman et al., 2013) which fuel the progression of airway wall remodeling (Boxall et al., 2006).

**Figure 1:**
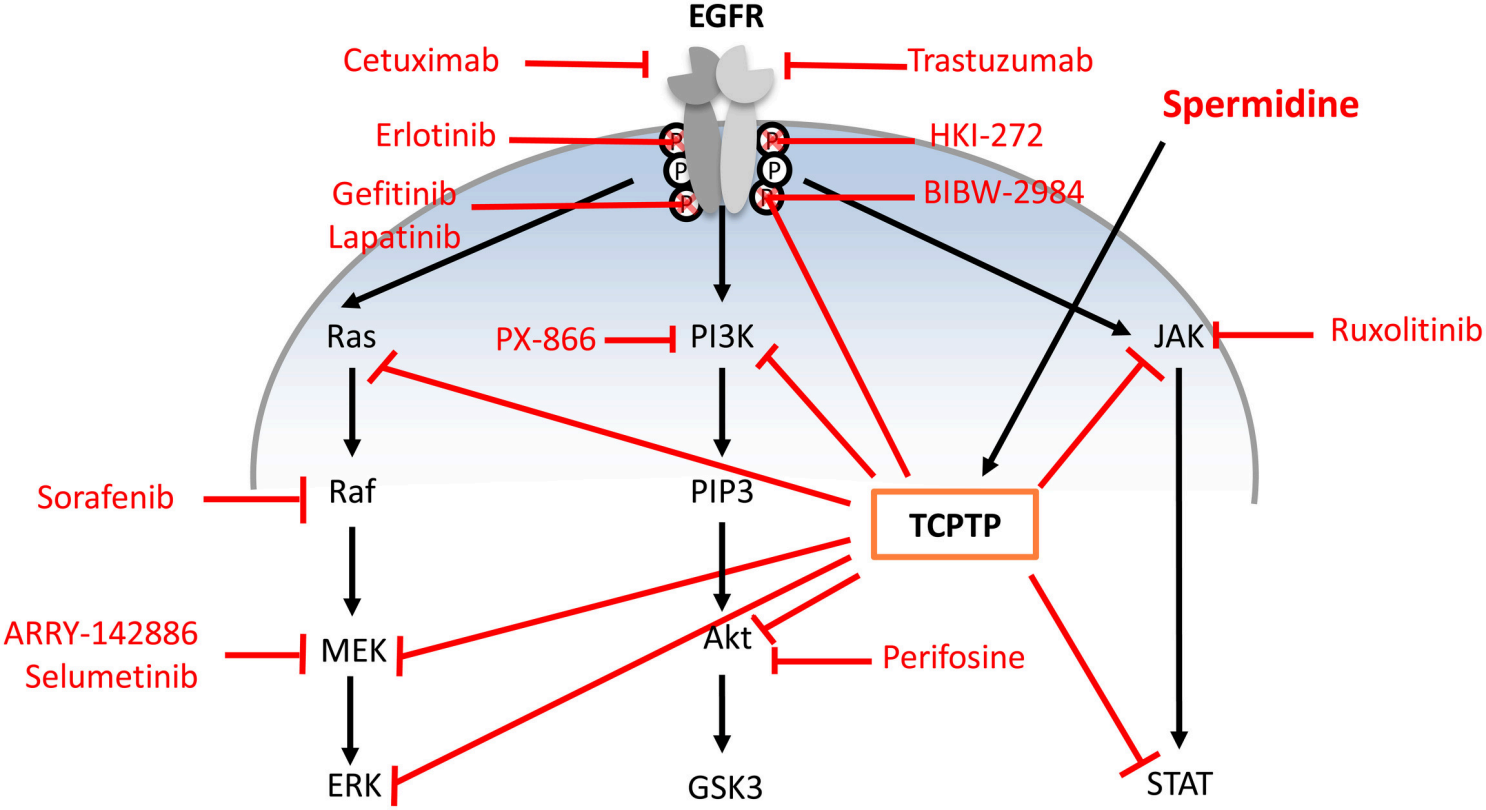
**Single-step pharmacologic strategies in chronic respiratory disease of approved and under-development EGFR pathway inhibitors compared to spermidine activity**. Single step strategies: (i) anti-EGFR monoclonal antibodies that bind the extracellular domain of EGFR and prevent downstream activation (cetuximab, trastuzumab); (ii) tyrosine kinase inhibitors targeting the intracellular portion of EGF receptors to block autophosphorylation (erlotinib, gefitinib, lapatinib, HKI-272, BIBW-2948); (iii) Inhibitors of specific downstream EGFR signaling pathway (ARRY-142886, PX-866, ruxolitinib, perifosine, selumetinib, and sorafenib). Acting on TCPTP, spermidine can exert multiple direct and indirect effects.

Impaired airway epithelial integrity also parallels the higher expression of epidermal growth factor receptor (EGFR) in airway epithelia (Burgel and Nadel, 2008). Dimeric EGFR cooperates with TGF-β in activating one or more downstream effectors, including the MEK/ERK (MAPK kinase/extracellular signal-regulated kinase), PI3K/AKT (phosphatidylinositol-3-kinase/protein kinase B), and STAT (signal transducer and activator of transcription) pathways through receptor autophosphorylation and cytoplasmic protein binding (Jorissen et al., 2003). Therefore, EGFR and its downstream signaling effectors have been targeted by a number of new drug candidates (Figure 1).

Many metabolites are involved in airway remodeling in obstructive diseases, e.g., the natural polyamines (PA) spermidine and spermine, although their role as either worsening or alleviating factors is complex and still under debate (Hoet and Nemery, 2000; Erle and Sheppard, 2014). In fact, the EMT transition and barrier disruption that are present in airway diseases parallel PA/spermidine scarcity (Prunotto et al., 2010). Conversely, spermidine alone may be able to moderate most consequences triggered by EGFR and its downstream effectors *via* agonism on T-cell protein tyrosine phosphatase (TCPTP; Figure 1), as better detailed hereafter.

## Role of polyamines in lung cell physiology

**Figure 2:**
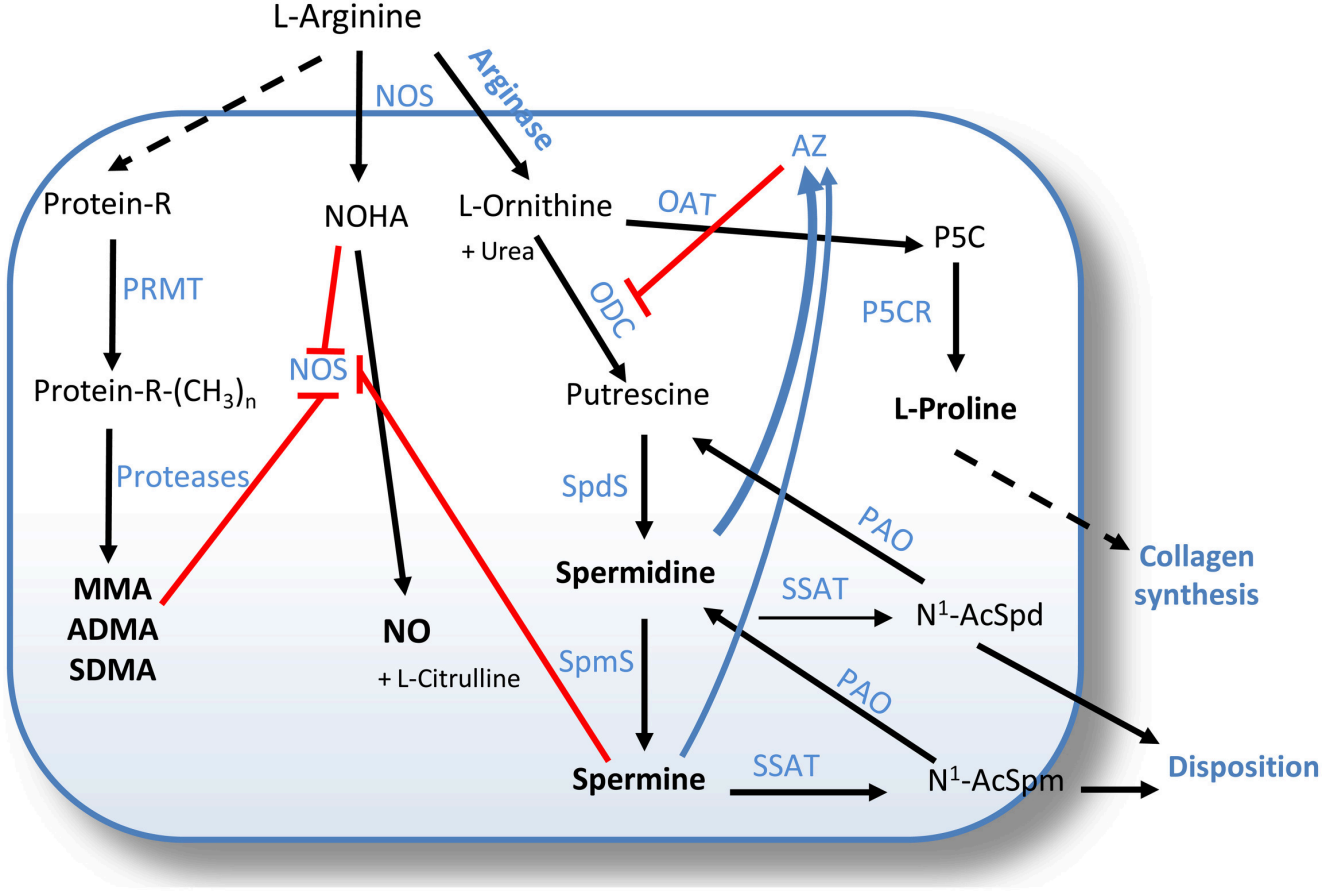
**Competing pathways in L-arginine metabolism**. Arginine is a substrate for both NOS, yielding NO and L-citrulline, and for arginase, to produce ornithine and urea. Ornithine is then metabolized by ODC to putrescine, or by OAT to provide proline, a major precursor for collagen biosynthesis. Putrescine supplies the building blocks of the higher polyamines spermidine and spermine, synthetized by SpdS and SpmS, respectively. ODC activity is controlled by AZ, which in turn accumulates via mechanisms triggered by increasing polyamine levels. The polyamine pool is also back-regulated via acetylation and degradation by SSAT and PAO. Both spermine and, to a lesser extent, spermidine, inhibit NOS activity. NOS inhibition can further derive from the feedback effect of NOHA, the intermediate in NO production. Arginine is also incorporated into proteins, where it can be mono- or di-methylated by PRMT to free MMA, ADMA, and SDMA upon proteolysis. ADMA acts as an endogenous inhibitor of NOS, and SDMA competes in L-arginine uptake. AZ, antizyme; NO, nitric oxide; NOS, nitric oxide synthase; NOHA, Nω-hydroxy-L-arginine; PRMT, protein arginine methyltransferases; Protein-R-(CH_3_)_*n*_, n-methyl-arginine-containing proteins; MMA, monomethyl arginine; ADMA, asymmetric dimethylarginine; SDMA, symmetric dimethylarginine; OAT, ornithine aminotransferase; ODC, ornithine decarboxylase; P5C, L-pyrroline-5- carboxylate; P5CR, pyrroline-5-carboxylate reductase; SpdS, spermidine synthase; SpmS, spermine synthase; PAO, polyamine oxidase; SSAT, spermidine/spermine-N1-acetyltransferase; N1-AcSpd, N1-acetylspermidine; N1-AcSpm, N1-acetylspermine.

Spermidine and spermine are the most common and important members of PA, small organic polycations ubiquitously present in nature. Spermidine [NH_2_(CH_2_)_4_NH(CH_2_)_3_NH_2_] and spermine [NH_2_(CH_2_)_3_NH(CH_2_)_4_NH(CH_2_)_3_NH_2_] are sequentially derived from putrescine [NH_2_(CH_2_)_4_NH_2_]. Their biosynthesis requires decarboxylated S-adenosyl-L-methionine (DcAdoMet) as aminopropyl group donor and the enzymatic activity of spermidine synthase and spermine synthase, respectively. PA are essential for living cells, where they specifically interact with DNA, RNA, histones, and other proteins, thus affecting gene expression and biological protein activity (Pegg, 2009; Igarashi and Kashiwagi, 2010). Each PA has an array of distinctive properties. A unique role of spermidine in eukaryotes is the covalent modification of eukaryotic initiation factor 5A (eIF5A), resulting in an unusual amino acid, hypusine [N^ϵ^-(4-amino-2-hydroxybutyl)lysine] (Park, 2006). eIF5A and modified hypusine are necessary for the viability and growth of mammalian cells (Nishimura et al., 2012). PA are synthesized by a pathway involving L-arginine metabolism *via* arginase and ornithine decarboxylase (ODC) and their synthesis is regulated through a limitative feed-back control activated by increased PA level through antizyme (AZ)-mediated ODC down-regulation (Figure 2). However, L-arginine, *via* the nitric oxide synthase (NOS) pathway, also supports the formation of NO, which is key for the maintenance of airway tone (Ray et al., 2014). The balance between NOS isozymes and arginases regulates airways tone (Meurs et al., 2003). In particular, the constitutive neuronal and endothelial isoforms (nNOS and eNOS) support lung smooth muscle tone and relaxation. Noteworthy, NO scarcity may be compensated by the presence of inducible NOS (iNOS), which is upregulated in airway inflammation, e.g., in asthma (North et al., 2010; North and Scott, 2011). A negative role for PA in lung diseases was suggested by studies showing that an increase in the arginase/PA pathway at the expense of the NOS/NO output exacerbates COPD and asthma (Bergeron et al., 2007). Actually, enhanced arginase activity decreases L-arginine availability, thereby causing NO deficiency that contributes to airway hyper-responsiveness (Morris, 2013; North et al., 2013). Following the suggestion that the NOS-to-arginase shift may play a negative role in airway remodeling (Meurs et al., 2002, 2003), PA biosynthesis has been proposed as a new therapeutic target (Pera et al., 2014).

The hypothesis that envisages a pathogenic role for PA is also supported by the findings of a study describing the integrin-mediated localization of spermidine/spermine-N1-acetyltransferase (SSAT, the key PA catabolic enzyme) in close proximity to the lipid kinase phosphatidylinositol phosphate (PIP)5K1γ, the main source of phosphatidylinositol 2 phosphate in airway smooth muscle, whose activity is regulated by PA. It has been shown that proper assembly of the integrin-SSAT-PIP5K1γ complex results in efficient PA catabolism and airways contraction inhibition, whereas administration of the higher-order PA spermine enhanced contraction (Chen et al., 2004, 2012). However, it should be noted that these studies used exposure doses (100 μM) at least 10-fold higher than physiologic levels of PA. It is also important to consider that exogenously added PA—even at low doses—can actually inhibit intracellular PA biosynthesis by negative feed-back, as mentioned above, possibly resulting in increased availability of L-arginine and its shift toward NO formation.

A further element of complexity derives from the occurrence of L-arginine derivatives, such asymmetric and symmetric dimethyl arginine (ADMA and SDMA, respectively) which represent degradation products of proteins containing post-translationally methylated arginine residues (Scott et al., 2011, 2014; Holguin et al., 2013; Scott and Grasemann, 2013). The accumulation of both ADMA and SDMA can lead to reduced NOS activity. ADMA is a NOS endogenous inhibitor associated with lung disease (Zakrzewicz and Eickelberg, 2009), whereas SDMA contributes to the NO imbalance by competing with the cationic amino acid transporter-2 for L-arginine uptake (North and Scott, 2011). ADMA levels range between 50 and 75 nM, whereas its potency in NOS inhibition is 100-fold higher than that of spermine. Hence, lung diseases such as acute respiratory dysfunction triggered by NO scarcity might be the result of proteolytic waste of L-arginine and subsequent accumulation of these arginine derivatives, rather than derive from PA production favored by the NOS-to-arginase shift.

## The key driving force in epithelial stabilization: TCTPT agonism

Although some of the studies presented above suggest a negative role for PA in airways diseases, we propose that one specific PA, i.e., spermidine, may play a positive (possibly therapeutic) role in pulmonary disarray by virtue of two mechanisms: interaction with TCPTP and activation of autophagy.

**Figure 3:**
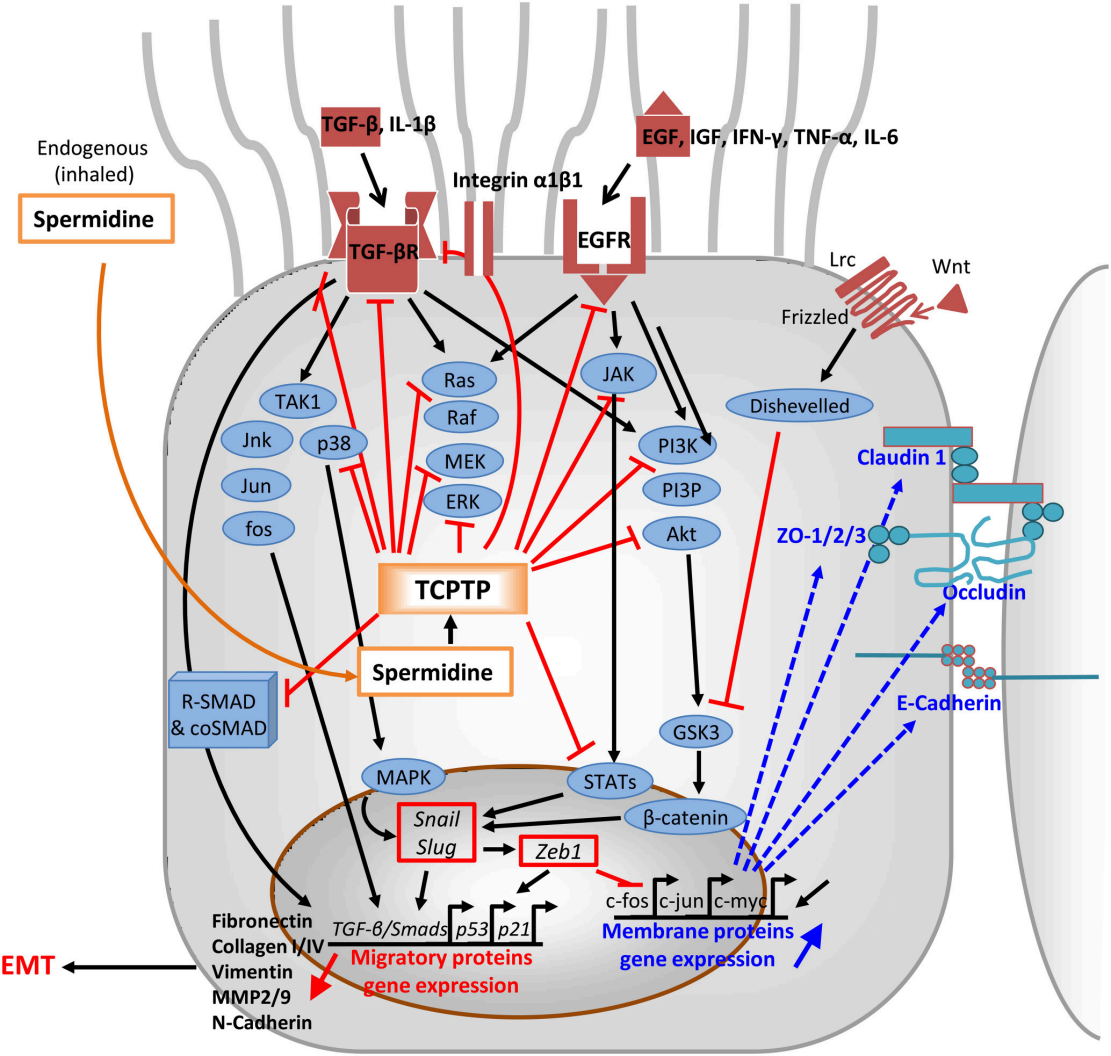
**Mode of action of spermidine through TCPTP-activation**. In the cytoplasm, activated TCPTP down-regulates signal transduction pathways downstream EGFR, TGF-βR, the Frizzled/Wnt/Lrc complex, and most of conjugated signaling effectors of EGF, IFN-γ, TNF-α, IL-6, and IL-1β/TGF-β. Inhibition occurs by dephoshorylation of key components of JAK1/jnk/jun/fos/p38, noncanonical Ras/Raf/MEK/ERK, and PI3K/PIP3/Akt pathways, as well as the nucleus-addressably proteins STATs and R-SMADs. In particular, dephosphorylated R-SMADs prevent coSMAD binding and the R-SMAD/coSMAD complexes to accumulate in the nucleus and act as transcription factors in TGF-β/Smads and p53 loci via Snail/Slug and Zeb1. Their inhibited access to chromatin represses detachment, migration, and EMT proteins like fibronectin, vimentin, MMP2/9, N-cadherin, and collagens I/III. Phenotypic involution toward EMT is prevented, whereas spermidine epigenetically favors the expression of c-fos, c-jun, and c-myc. Outcomes consist of the enhanced expression of tight junctional and adherens proteins: claudin-1, occludin, ZO-1/2/3, and E-cadherins (in bold). EGF, epidermal growth factor; EGFR, EGF receptor; TGF-β, transforming growth factor β; TGF-βR, TGF-β receptor, TGFβRI/II, TGFR tetramer; TAK1, TGFβ-activated kinase 1; IFN-γ, interferon-γ; TNF-α, tumor necrosis factor-α; IL-6, interleukin-6;, IL-1β, interleukin-1β; Wnt/Lrc, ligand to Frizzled receptor; Jnk/Jun/fos/p38 signal transduction proteins; ERK, extracellular signal-regulated kinase; PI3K, phosphatidylinositol-3'- kinase; PIP3, phosphatidylinositol(3,4,5) trisphosphate; Akt, activator kinase; JAK, Janus kinase; R-SMAD and coSMAD, family of transcriptional smadx activator protein/cofactors; STATs, signal transducers/activators of transcription; GSK3, glycogen synthase kinase; ZO, zonula occludens; Snail/Slug, transcription factors; Zeb, zinc finger E box binding homeobox; TGFβ/Smads/p53/p21, genes for chemotaxis; c-fos/c-jun, gene expressing junctional proteins; MMP, matrix metalloproteinases; EMT, epithelial-to-mesenchymal transition.

TCPTP *(aka* PTPN2), a phosphatase expressed in most mammalian cells, is an intracellular suppressor of metastatic and inflammatory signaling pathways. The two TCPTP isoforms, resulting from differential splicing, suppress a variety of signaling substrates including receptor tyrosine kinases (RTKs), such as EGFR, insulin growth factor receptor (IGFR), platelet-derived growth factor receptor (PDGR), colony-stimulating factor-1 (CSF-1) receptor, and vascular endothelial growth factor receptor (VEGFR), as well as mitogen-activated protein kinases (MAPKs). Moreover, TCPTP quenches signaling mediated by nuclear (e.g., STAT-1, -3, -5, 6), and cytoplasmic proteins (e.g., JAK1, JAK2, p52-Shc, Src-tyrosine kinases; Mattila, 2009; Muppirala et al., 2013). The dephosphorylation of key targets in downstream signaling pathways leads to the inactivation of pro-inflammatory responses induced by IFN-γ, TNF-α, and IL-6. TCPTP proteins impact airway physiology in multifarious ways (Figure 3), mostly by quenching TGF-βR and EGFR (Tiganis et al., 1999; Mattila et al., 2005) and relevant post-receptor signaling intermediates, thus providing an anti-mitogenic effect (Chernoff, 1999; Rajeeve et al., 2013).

Recently, a high-throughput assay aimed at selecting novel TCPTP agonists screened as many as 64,280 small molecules to identify finally two leads, mitoxantrone and spermidine, both active at low micromolar levels (Mattila et al., 2010). Modalities of action were elucidated: the former directly binds to TCPTP catalytic domain, whereas spermidine agonism does not require interference with TCPTP active center (Ylilauri et al., 2013).

This study showing a direct interaction between spermidine and TCPTP may provide a unifying explanatory view of a vast array of spermidine biological features, firstly described in human dermal fibroblasts and still lacking an explanation, such as the down-regulation of matrix metalloproteinase-2, histone acetyltransferase, p-ERK, p-JNK, and NF-κB activity and expression, and increase in histone deacetylase 1 and sirtuin 1 (Park and Kim, 2012).

Indeed, parallel studies confirmed that the increase of TCPTP level and enzymatic activity driven by spermidine were coupled with decreased phosphorylation of the downstream transducers in IFN-γ cascades (Penrose et al., 2013). TCPTP activation in response to stress factors was also observed in human THP-1 monocytes challenged with IFN-γ. Exogenous spermidine enhanced TCPTP level, reduced phosphorylation of STAT-1,3, p38-MAPK, and lowered secretion of ICAM-1, monocyte chemoattractant protein (MCP)-1, and IL-6 in a TCPTP-dependent manner, as the modulatory effects of spermidine against IFN-γ exposure were absent in TCPTP-knockdown cells (Moron et al., 2013).

## Lessons from the intestinal epithelium

The connection of PA with intestinal repair, replacement and stabilization has been investigated for 40 years (Luk and Yang, 1987; Wang and Johnson, 1990, 1992; McCormack and Johnson, 1991; Johnson and McCormack, 1999; Timmons et al., 2012). Several studies have evaluated their effect on the growth capacity of the gastrointestinal mucosa, which has the highest turnover in human body (approximately every 78 h), and have shown a positive role for PA both in conditions of physiological growth and in mucosal repair. In fact, lack of PA stabilizes p53 and JunD, thereby inhibiting intestinal epithelial re-growth (Li et al., 2001, 2002). In addition, since appropriate PA levels are essential for the stability of occludin and adherens junctions (Guo et al., 2003, 2005), as well as for c-myc-mediated E-cadherin transcription (Liu et al., 2005, 2009), PA might have a beneficial effect in maintaining the intestinal epithelial barrier function. This “improved barrier” theory has a sound background since PA, and particularly spermidine, assist cell-cell interactions by promoting the expression of adherens and tight junction proteins in the intestine (Wang, 2005).

Another mode of action of PA in ensuring mucosal integrity was revealed by a study showing that PA are needed for microtubule (MT) formation during gastric mucosal healing (Banan et al., 1998). These results are in line with the essential role of PA in cytoskeleton assembly (Anderson et al., 1985). In this context, the facilitated tubulin attraction, MT nucleation, and elongation promoted by PA, and particularly by spermidine, occur through plain electrostatic mechanism (Mechulam et al., 2009; Hamon et al., 2011). Indeed, PA depletion causes the disappearance of actin filaments and MT in CHO cells (Pohjanpelto et al., 1981), while PA was found to associate to anionic MT *via* promoted formation of MAP4 (MT-associated protein 4) and redistribution of EB1 (end-binding protein 1) and MT plus-end-binding protein (Savarin et al., 2010). Therefore, as gap junctions require extended MT up to cell periphery (Shaw et al., 2007), it is possible to envisage a process in which PA-induced MT elongation and modulation sustain epithelial integrity.

In these settings, TCPTP agonism enacted by spermidine matches 40 years of research on bowel homeostasis, and also explains the molecular mechanisms underlying the contribution of spermidine to the alleviation of pro-inflammatory signals and intestinal barrier dysfunction. In fact, on a functional level, it has been shown that spermidine in co-incubation experiments with IFN-α protects intestinal epithelial barrier functionality in the setting of inflammation, by limiting the increase of epithelial permeability (Penrose et al., 2013). Moreover, spermidine administration decreased mucosal damage and weight loss in a mouse model of colitis (Moron et al., 2013).

Finally, the anti-“inflammaging” action and barrier reinforcement triggered by TCPTP agonism (or autophagy, see below) may account for the positive effects of enhanced spermidine/PA production in the luminal tracts on longevity (Matsumoto et al., 2011; Kibe et al., 2014) and tumorigenesis (Soda et al., 2013).

## Autophagy: promotion by spermidine and role in lung disorders

**Figure 4:**
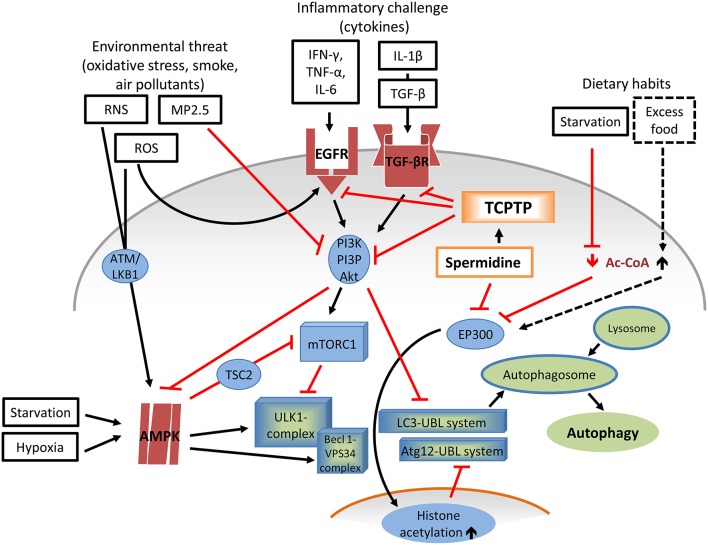
**Role of Spermidine-TCPTP in the complex dynamics of pro- and anti-autophagic factors**. Autophagy requires the coordinated activation of multiple factors. Several Atg proteins are recruited within the ULK1 complex during vesicle nucleation and within the ubiquitin-like conjugation systems Atg12-UBL and LC3-UBL during phagophore elongation, and completion. The main autophagy repressor, mTORC1, exerts its inhibitory action by phosphorylating ULK1. mTOR interacts with the proline-rich Akt, newly synthesized in the PI3K/PI3P/Akt pathway, downstream of EGFR, and TGF-βR signaling, which in turns responds (inter-alia) to cytokines such as IFN-γ, TNF-α, IL-6, and IL-1β/TGF-β. A mTORC1 antagonist and autophagy promoter is AMPK. Upon activation from non-immune stress factors including starvation, hypoxia, ROS (e.g., ${\text{O}}_{2}^{-}$, H_2_O_2_, OH^.^), and RNS (e.g., peroxynitrite) via ATM/LKB1, AMPK prompts autophagy. AMPK is directly involved in the formation of ULK1 complex and cooperates with Becl 1-VPS34 complexes, pro-autophagy adapters involved in nucleation of autophagosome membrane. While ROS simulate EGFR activation, other environmental stressors (e.g., MP2.5) have an opposite effect, by inhibiting PI3K/PI3P/Akt. Agonistic activity of spermidine on TCPTP would down-regulate both EGFR and TGF-βR and their downstream effectors. Yet, the prevalent, established mode of action of spermidine in autophagy entails the inhibition of EP300, leading to reduced histone acetylation (particularly histone-3), and, consequently, to Atg (particularly Atg7) expression and autophagosome elongation and closure. Spermidine activity on EP300 is similar to that exerted by low cytoplasmatic levels of Ac-CoA due to scarce nutrient availability, a situation that also activates AMPK. Ac-CoA, acetyl-coenzyme A; ROS, reactive oxygen species; RNS, reactive nitrogen species; ${\text{O}}_{2,}^{-}$ superoxide anion; H_2_O_2_; hydrogen peroxide; OH^.^, hydroxyl radical; PM2.5, particulate matter 2.5; IFN-γ, interferon-γ; TNF-α, tumor necrosis factor-α; IL-6, interleukin-6;, IL-1β, interleukin-1β; EGFR, epidermal growth factor receptor; TGF-βR, transforming growth factor β receptor; ULK1/2, UNC-51-like kinases 1/2; ATM ataxia telangiectasia mutated kinase; LKB1, upstream kinase of AMPK; AMPK adenosine monophosphate-activated protein kinase; TSC2, tuberous sclerosis 2; mTORC1, mammalian target of rapamycin type-1 complex; EP300, E1A-binding protein p300; Beclin 1-VPS34 complex, Beclin 1-ATG14L; VPS34, class III phosphatidylinositol 3-kinase; Atg autophagy related proteins; ULK1-complex, Atg13-FIP200-Atg101-Atg14L conjugation system; FIP200, FAK-family interacting protein of 200 kDa; Atg12-UBL system, Atg5-Atg12-Atg10-Atg16-Atg7 pathway; LC3-UBL system, LC3-Atg3-Atg4-Atg7-PE pathway; LC3, microtubule-associated protein light chain 3; PE, phosphatidylethanolamine; HAT, histone acetyltrasferase.

Autophagy is a self-cleaning process that represents an adaptive response elicited by “anti-nutrient” signals and starvation, and contributes to longevity (Eisenberg et al., 2009). Its role in COPD and asthma is complex and not fully clear (Poon A., et al., 2012; Poon A. H., et al., 2012; Jyothula and Eissa, 2013). Among the factors involved, oxidative stress may act both as autophagy promoter via AMP-activated protein kinase (AMPK) signaling (Wu et al., 2014), and repressor, in analogy with cytokine effectors that dock EGFR and TFG-βR (Khan et al., 2008; Tripathi et al., 2013). Conversely, particulate matter 2.5 (PM2.5), an aggravating factor for most obstructive airways diseases, seems to favor autophagy, as it inhibits the PI3K/AKT/ mammalian target of rapamycin type 1 (mTOR) pathway (Liu et al., 2015). Overall, the overactive immune responses present in airway pathologies display an array of divergent pro- and anti-autophagy effects (Figure 4).

As noted by Mercado et al. (2015), current information about the role of autophagy in bronchial and alveolar epithelial cells in lungs of patients with active COPD is divided into evidence showing that excessive autophagy and mitophagy results in cell death, and proof that pathological changes are the consequence of an underlying defective autophagy. Insufficient autophagy is thought to lead to involution patterns including stem cells depletion, reduced antioxidant responses, and defective mitochondrial function, which may contribute to progressive cell senescence (Fujii et al., 2012; Ito et al., 2012; Takasaka et al., 2014). Conversely, excess autophagy leads to shortening and, finally, total consumption of cilia components, called “ciliophagy” (Lam et al., 2013). Epithelial cells from mice lacking LC3B, which is required for autophagosome formation, are protected from cigarette-smoke (CS)-induced autophagy and resist emphysema. However, the effect of CS-promoted ciliophagy on mucociliary clearance is reversible and only lately associated with the increased apoptosis of ciliated cells that occurs when accumulation of CS-denatured protein exceeds cellular degradative capacity. This suggests that ciliophagy only partially contributes to airway dysfunction (Cloonan et al., 2014) and might be a regulatory pattern in ciliogenesis through the control of ciliary proteins (Orhon et al., 2015).

In clinical specimens of COPD patients, autophagy markers are upregulated at early stages of disease progression whereas caspases are activated later on. This might indicate that autophagy either precedes apoptosis in response to disease stress on lung, or is an attempt to self-protect from CS stress (Ryter et al., 2009). Autophagy may altogether act as a clearance mechanism, since macrophages in lung biopsies from non-COPD smokers showed impaired autophagic efficiency and debris accumulation (Monick et al., 2010).

With regard to the protective role of spermidine in airway diseases, it is of interest to underline that the promotion of authophagy is another key aspect of spermidine activity (Eisenberg et al., 2009; Morselli et al., 2011; Minois, 2014). The beneficial impact exerted by spermidine in several model systems, including improved fertility (Bauer et al., 2013) and retained memory in aging (Gupta et al., 2013) or reverted age-dependent arterial degeneration in murine arteriosclerosis (LaRocca et al., 2013), correlates with the activity of autophagy promotion. Outcomes are comparable to starvation and caloric restriction mimetics, a new class of autophagy-inducing therapeutics with potential clinical utility in diabetes, metabolic syndrome, and cardiovascular diseases (Madeo et al., 2014). Autophagy is repressed by mTOR complex (mTORC1), but is activated by pathways involving the “longevity factor” Sirt1 or the “energy sensor” AMPK (Cetrullo et al., 2015). However, spermidine appears to function as an autophagy inducer not by directly affecting the mTOR pathway, but rather by inducing a status of protein hypoacetylation via inhibition of histone acetylases (Morselli et al., 2009), such as EP300 acetyltransferase, an endogenous repressor of autophagy (Pietrocola et al., 2015).

As EP300 is strictly regulated by cytoplasmatic acetyl-coenzyme A levels (Marino et al., 2014b) and nutrient availability, this confirms the “starvation-mimicking” property of spermidine (Figure 4).

In line with the proposed role of the crosstalk between spermidine and TCPTP (see above), recent studies have demonstrated that TCPTP also regulates autophagy in human intestinal cells and other cell types (Scharl and Rogler, 2012; Scharl et al., 2012). TCPTP dysfunction correlated with impaired autophagosome formation and defective autophagy, thus contributing to the development and perpetuation of chronic inflammatory disorders, such as inflammatory bowel diseases (Spalinger et al., 2015). Conversely, high TCPTP levels were found to increase autophagy (Aradi et al., 2015). Therefore, it may be speculated that spermidine can promote the autophagic process at least in part via TCPTP; however, the role of TCPTP in spermidine-induced autophagy remains to be explored, together with a careful examination of the effective dose. Indeed, manipulation of autophagy is a delicate matter, as this process can generally antagonize degenerative processes, but its excessive induction may result in maladaptive tissue remodeling (Fimia and Piacentini, 2010; Marino et al., 2014a; Filomeni et al., 2015; Maiuri and Kroemer, 2015). Thus, it is important to monitor carefully basal levels of autophagy and its flux as a function of time and dosage of stimulant. This can be carried out with experimental models usually employed to study chronic epithelial disorders, such as *in vitro* cultures of immortalized human bronchial epithelial cell line (BEAS 2B cells) or primary cells from specimens obtained from donors for lung transplantation (Kinnula et al., 1994). Genetic deletion of specific autophagy proteins in animal models (Cann et al., 2008) may also be useful to study the involvement of this process in airway disorders and the possible modulation by spermidine treatment. Keeping in mind the complexity of the autophagic process, multiple, short-term and long-term assays are recommended to monitor autophagy, including flux measurements in normal, and blocked turnover conditions. Indeed, autophagy flux is the entire process, from autophagosome biogenesis to cargo digestion by lysosomes, and macromolecule release back into the cytosol. Therefore, the evaluation of lysosomal competence as the final stage in this process is essential for a correct interpretation of the results. Finally, human lung specimens from patients can be analyzed for autophagy markers, such as increased autophagosome numbers and increased expression of LC3B-II, the active form of LC3B, or assayed by transmission electronic microscopy to monitor autophagic structures. This issue, i.e., the different outcome of autophagy as a function of its level, introduces dose-finding concepts in pursuit of the therapeutic use of exogenous spermidine, and underscores the need for fine-tuning the dose of spermidine in clinical settings, as further detailed in the next paragraph.

## Therapeutic applications

Spermidine is currently used as dietary supplements and topical hair-growth promoter, with proven efficacy *in vitro* (Ramot et al., 2011) and in clinical settings (Rinaldi et al., 2004); therefore, the molecule has a proven history of safe long-term use in consumer products.

Over the past 5 years our group has set up a therapeutic platform based on spermidine. We have focused on the preparation and set up of a variety of supramolecular complexes (SMC) in which spermidine was bound to different polymers, and our studies showed that sustained delivery from SMC is able to optimize the effect of spermidine on fibroblast viability (Ghisalberti et al., 2013). The sustained-release efficiency is unrelated to the matrix polymer, since a variety of anionic or SMC-forming carriers (e.g., spermidine-hyaluronate, Spd-HA) afforded similar results.

A pilot study on vestibulitis, in which pain relief was assessed in terms of absolute and relative effect size compared to present and purported drugs for vulvodynia (Murina et al., unpublished), showed that a regimen of 1 μmole of spermidine (administered as gels containing Spd-HA) at 3 doses/week for 1 month, followed by 2 doses/week for another month was effective in blunting pain, assessed by the visual analogic score, and improving dyspareunia, thus indicating that spermidine may be envisaged as a new paradigm in widespread female disorders. Indeed, another pilot study in post-menopausal vaginal atrophy showed that a similar regimen, but by intravaginal application, resulted in clinical outcomes close to those of estrogenic and pro-estrogenic therapies (Carena Maini et al., unpublished), yet avoiding estrogenic risks while retaining comparable efficacy and duration. Other exploratory trials to evaluate the efficacy of spermidine complexed to SMC for atrophic or degenerative disorders, such as periodontitis and osteoarthritis are ongoing, following a series of studies on joint metabolism and mesenchymal differentiation (Borzi et al., 2014).

**Figure 5:**
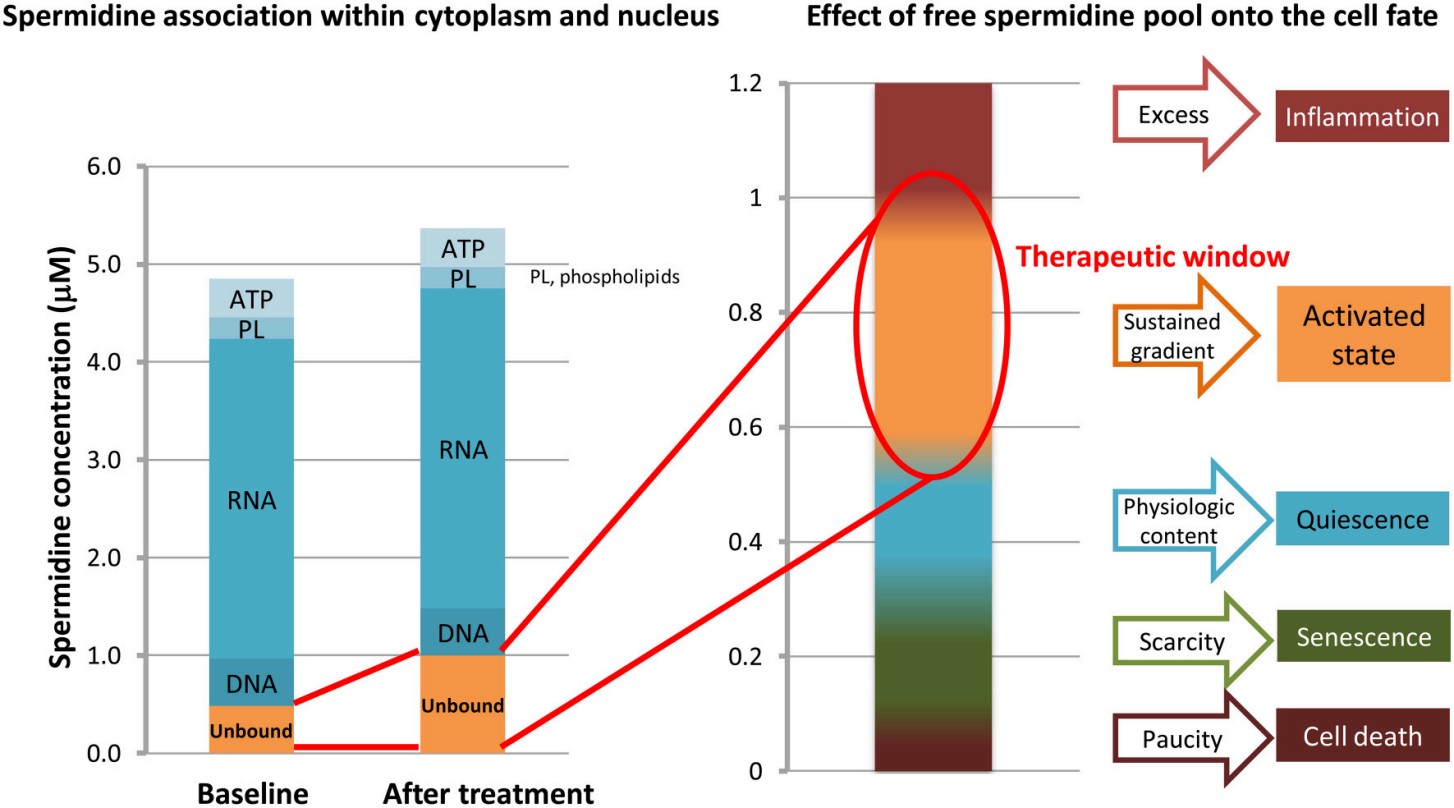
**Effects of unbound spermidine on cellular fate**. The multimodal activity of spermidine primarily relies on local concentration, recognized as a critical factor. The polycationic spermidine tends to saturate anionic molecules, partly in the phospholipid (PL) bilayer within cell membranes, partly coupling phosphate and adenosine triphosphate (ATP); yet, it is mostly associated with the core anionic biopolymers, i.e., RNA, but also DNA and histones, therefore modifying chromatin conformation. Ultralow doses of exogenous spermidine double the content of free (unbound) spermidine. Excess and paucity levels are closer than previously deemed, as a change in the micromolar range may switch a trophic (or growth promoting) activated state pattern into an inflammatory-apoptotic pattern. Concentrations are just indicatory, as levels of spermidine in cytosol and nucleus differ from tissues to tissues and during lifespan.

The question of “how a common metabolite at sub-micromolar dose becomes of therapeutic relevance” may have an answer in the localization of spermidine, which is 90% bound to intracellular anionic moieties (Watanabe et al., 1991; Matthews, 1993). Therefore, even a low dose in the micromolar range can lead to a meaningful increase, actually doubling the free pool and thereby impacting on overall cell physiology. Hence, spermidine “therapeutic window” may reside at sub-micromolar level (Figure 5). In addition, this dosage prevents plain toxicity (Foster et al., 1990) while impeding apoptotic/inflammatory cascades to prevail (Silva et al., 2011).

Interestingly, it has been shown that low doses of spermidine in the micromolar range are sufficient to activate TCPTP (Mattila et al., 2010), thereby triggering the downstream signaling pathways discussed above, and also activating autophagy, which has been shown to be induced by low doses of spermidine (Eisenberg et al., 2009). Activated TCPTP moderates most destabilizing processes set in motion by secondary growth and stress factors in asthma and COPD airways, while sustaining epithelial barrier repair and reinforcement. Moreover, the addition of spermidine should contrast increasing polyamine biosynthesis, which may negatively impact on NO formation. Although only preliminary data are presently available, it can be predicted and experimentally tested that sub-micromolar doses of spermidine in SMC, possibly delivered using an inhalator, may be beneficial in bronchial repair to improve patient outcomes in chronic lung diseases, including asthma and COPD.

## Conclusions

**Figure 6:**
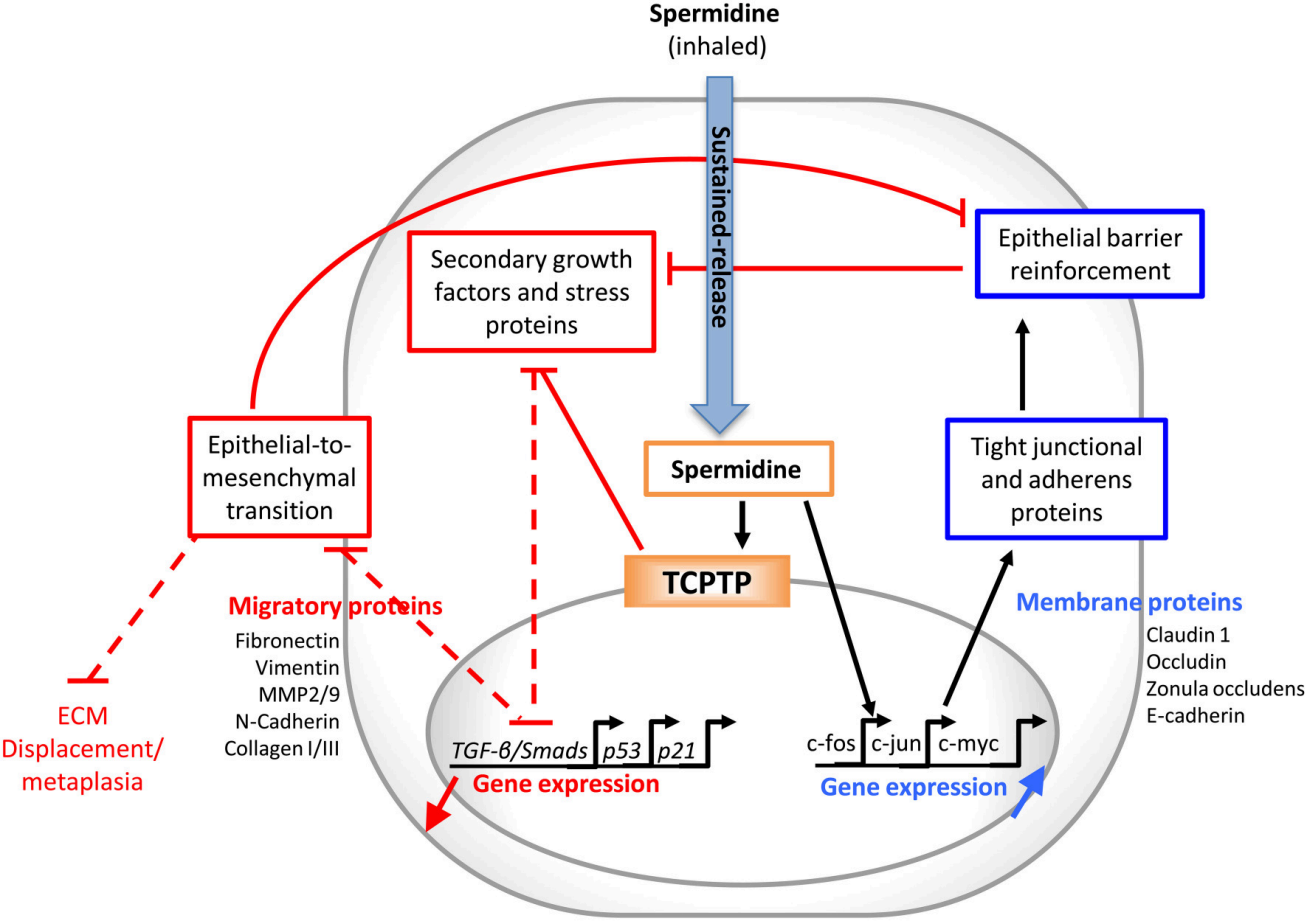
**Exogenous spermidine may interrupt the vicious cycling in chronic airways diseases**. Locally delivered spermidine works both as a TCPTP agonist and stimulator of the expression of genes coding for adherens and tight junction proteins. The former effect down-regulates the secondary growth factor signaling pathways, such as epidermal growth factor (EGF) and transforming growth factor-β (TGF-β) receptors and relevant pathways. This repression prevents the epithelial-to-mesenchymal transition to occur, which would further impair the epithelial impermeability and contribute to chronic remodeling and fibrosis. Strengthening the airways epithelial barrier translates into a reduced exposure to allergens, stressor proteins, and reactive oxygen species.

A reparative approach of disrupted epithelium in obstructive airway diseases may afford protection and more lasting results than therapies such as corticosteroids or immunosuppressant drugs. PA have been proposed as potential therapy in airway diseases. However, it has also been claimed that increased PA biosynthesis exacerbates lung diseases, as the PA synthetic pathway competes for L-arginine at the expenses of NO production, thus resulting in bronchoconstriction. Moreover, the pathways involved in the beneficial effect of PA in airway diseases are still poorly defined and controversial. A recent study showing a direct interaction between spermidine and TCPTP may place the beneficial role of PA in airway epithelial integrity in a coherent framework. In fact, the dephosphorylating activity of TCPTP represses the signaling cascade that activates genes favoring cell detachment and epithelial-to-mesenchimal transition, while increasing the expression of adhesion and tight junction proteins. Moreover, the protective effect of spermidine may also depend on the capacity of TCPTP to trigger autophagy. As such, TCPTP agonism may provide a unifying explanatory view of a vast array of biological features of spermidine and foster a mechanism-guided application of PA in COPD. Since, doses of spermidine in the micromolar range are sufficient to activate TCPTP and downstream signaling pathways, the sustained delivery of very low amounts of spermidine from SMC may provide an optimal pharmacologic profile for the treatment of obstructive airway diseases while avoiding potential harmful effects of excess PA, as suggested by preliminary evidence obtained *in vitro* and *in vivo*. Therefore, we propose spermidine in a sustained release modality as a novel therapeutic approach for airway barrier remodeling, based on a novel mode of action: TCPTP agonism. The overall features of inhaled spermidine (Figure 6) appear promising in order to provide hope for chronic lung sufferers, who are still in need of novel therapies providing more appropriate, long-lasting benefits to effectively treat chronic lung diseases.

## Author contributions

CAG conceived the review and co-wrote the paper; GC co-wrote the paper; SC, FF, and RMB substantially contributed to editing and writing the paper.

## Funding

This work was supported by grants from Ministero dell'Istruzione, dell'Università e della Ricerca, Italy, (PRIN and FIRB RBAP10KCNS), University of Bologna (RFO), and Ministero della Salute (Fondi cinque per mille).

## Disclosure

CAG owns shares in Tixupharma.

### Conflict of interest statement

The authors declare that the research was conducted in the absence of any commercial or financial relationships that could be construed as a potential conflict of interest.
